# Delirium screening, prevention and treatment in the ICU: a systematic review of implementation strategies

**DOI:** 10.1186/cc13613

**Published:** 2014-03-17

**Authors:** Z Trogrlic, M Van der Jagt, J Bakker, MC Balas, EW Ely, PH Van den Voort, E Ista

**Affiliations:** 1 Erasmus MC University Medical Center Rotterdam, the Netherlands; 2 The Ohio State University, Columbus, OH, USA; 3 Vanderbilt University Medical Center, Nashville, TN, USA; 4 Onze Lieve Vrouwe Gasthuis, Amsterdam, the Netherlands; 5 Erasmus MC University Medical Center Rotterdam - Sophia Children's Hospital, Rotterdam, the Netherlands

## Introduction

The occurrence of delirium heralds a circumstance of higher risk of death, longer stay, higher cost, and greater likelihood of long-term brain dysfunction, and yet the majority of ICU patients worldwide do not get routinely monitored for delirium, thus obstructing timely prevention and management strategies. Determination of effective implementation strategies regarding screening, treatment and prevention of delirium is critical to address needed modifications in the culture of patient care in the ICU. The aim of this study was to summarize the efficacy of and the barriers to implementation of delirium management.

## Methods

We searched PubMed, Embase, PsychINFO, Cochrane and CINAHL for studies published between January 2000 and October 2012 and included them when implementation strategies and their efficacy and/or potential barriers of implementation were described.

## Results

In all studies (*n *= 34) multifaceted education strategies were used and combined (median: 5; IQR: 4 to 6). Positive results were reported for both process and clinical outcomes: adherence to delirium screening - improvement after implementation compared with the before measurement, by 15 to 57%; only measured after implementation, between 84 and 92%; delirium knowledge (on a 10-point scale; improved from 6.1 and 6.2 before to 8.2 and 7.4 respectively (*P *< 0.001) after implementation); length of stay in the ICU (4.1 vs. 5.9 days; *P *= 0.21 and 6.3 to 5.35 days; *P <*0.009) and hospital stay (12 days vs. 18 days; *P *= 0.036 and from 55 to 27 days; *P *< 0.0001); and decreased mortality (29.4% vs. 22.9%; *P *= 0.009; and OR = 0.45; 95% CI = 0.22 to 0.92; *P *= 0.03). The major barriers found impairing implementation concerned clinicians' attitude and healthcare professionals' knowledge.

## Conclusion

Implementation strategies used suggest a strong potential for multifaceted strategies to be able to affect both process and clinical outcomes. The majority of studies focused only on implementation of delirium screening. There is a knowledge deficit regarding efficacy of well-prepared implementation of integral delirium management considering screening, prevention, and treatment preceded by analysis of barriers. Further research on the extent and contents of implementation interventions aimed at integral delirium management such as those described in the recent ABCDE bundle and PAD guideline are necessary.

